# Herpesvirus infections and Alzheimer’s disease: a Mendelian randomization study

**DOI:** 10.1186/s13195-021-00905-5

**Published:** 2021-09-24

**Authors:** Shu-Yi Huang, Yu-Xiang Yang, Kevin Kuo, Hong-Qi Li, Xue-Ning Shen, Shi-Dong Chen, Mei Cui, Lan Tan, Qiang Dong, Jin-Tai Yu

**Affiliations:** 1 Department of Neurology and Institute of Neurology, Huashan Hospital, State Key Laboratory of Medical Neurobiology and MOE Frontiers Center for Brain Science, Shanghai Medical College, Fudan University, 12th Wulumuqi Zhong Road, Shanghai, 200040 China; 2 Department of Neurology, Qingdao Municipal Hospital, Qingdao University, 266071 Qingdao, China

**Keywords:** Herpesvirus, Infection, Alzheimer’s disease, Mendelian randomization

## Abstract

**Background:**

Observational studies have suggested that herpesvirus infection increased the risk of Alzheimer’s disease (AD), but it is unclear whether the association is causal. The aim of the present study is to evaluate the causal relationship between four herpesvirus infections and AD.

**Methods:**

We performed a two-sample Mendelian randomization analysis to investigate association of four active herpesvirus infections with AD using summary statistics from genome-wide association studies. The four herpesvirus infections (i.e., chickenpox, shingles, cold sores, mononucleosis) are caused by varicella-zoster virus, herpes simplex virus type 1, and Epstein-Barr virus (EBV), respectively. A large summary statistics data from International Genomics of Alzheimer’s Project was used in primary analysis, including 21,982 AD cases and 41,944 controls. Validation was further performed using family history of AD data from UK Biobank (27,696 cases of maternal AD, 14,338 cases of paternal AD and 272,244 controls).

**Results:**

We found evidence of a significant association between mononucleosis (caused by EBV) and risk of AD after false discovery rates (FDR) correction (odds ratio [OR] = 1.634, 95% confidence interval [CI] = 1.092–2.446, *P* = 0.017, FDR-corrected *P* = 0.034). It has been verified in validation analysis that mononucleosis is also associated with family history of AD **(**OR [95% CI] = 1.392 [1.061, 1.826], *P* = 0.017). Genetically predicted shingles were associated with AD risk (OR [95% CI] = 0.867 [0.784, 0.958], *P* = 0.005, FDR-corrected *P* = 0.020), while genetically predicted chickenpox was suggestively associated with increased family history of AD (OR [95% CI] = 1.147 [1.007, 1.307], *P* = 0.039).

**Conclusions:**

Our findings provided evidence supporting a positive relationship between mononucleosis and AD, indicating a causal link between EBV infection and AD. Further elucidations of this association and underlying mechanisms are likely to identify feasible interventions to promote AD prevention.

**Supplementary Information:**

The online version contains supplementary material available at 10.1186/s13195-021-00905-5.

## Background

The possibility of an infectious etiology for Alzheimer’s disease (AD) has long been suspected, including the roles of viruses, bacteria, and parasites. Recent meta-analyses have investigated and suggested that some herpesvirus infections were associated with a higher risk of AD [1, 2], especially the infection of human herpes virus-1 (HSV-1), human herpes virus-6, and Epstein-Barr virus (EBV). However, the current observational studies are limited by residual, unmeasured confounding, or other biases such as reverse causation and detection bias [3, 4]. It is still unclear whether the associations are causal relationships [5]. Recently, an article has detected EBV-specific T cell receptors in cerebrospinal fluid from patients with AD, which were enhanced in increased antigen-specific clonal expansion of CD8 + T cells in AD [6]. Although this article implied an association between EBV infectivity and AD in a new perspective, their data were still not a direct evidence of causation.

Mendelian randomization (MR) is an analytic approach using genetic variants as instrumental variables (IVs) for an exposure. Like randomized control trials, MR analyses reduce confounding and reverse causality due to the random allocation of genotypes from parents to offspring [7]. MR analyses are increasingly used to determine causal effects between potentially modifiable risk factors and the outcomes. A previous MR analysis has highlighted that MR can be used as an initial screening tool for validating the association between infection and AD [8]. Although HSV have been reported to be associated with AD in many epidemiological studies [2, 9], Kwok et al. found no causal association of the HSV infection with cognitive function or late-onset AD using MR analysis [8]. Thus, it is necessary to investigate the potential causality between herpesvirus infections and risk of AD using an unbiased approach. What is more, two-sample MR analysis is an extension in which the effects of the genetic instruments on exposure and outcome are obtained from separate genome-wide association studies (GWAS) [10]. The recent large-scale genome-wide datasets of infections and AD enable us to link four herpesvirus infections (i.e., chickenpox, shingles, cold sores, and mononucleosis) with risk of AD using two-sample MR approach [11, 12].

The four herpesvirus infections involved in the present MR study have been reported to be linked with AD [1, 2], which are mainly caused by varicella-zoster virus (VZV), HSV-1, and EBV, respectively. Primary infection of these herpesviruses typically occurs at a young age. Each persists in latent form following resolution of the primary infection and can reactivate once again. Chickenpox results from primary infection of VZV in childhood, while shingles are caused by the reactivation of latent VZV in later life. Cold sores are mainly caused by reactivation of HSV-1. Over 90% of the world’s adult population is chronically infected with EBV. However, primary infections in children are usually asymptomatic. When primary EBV infection occurs later in life, it often results in mononucleosis [13].

In the present study, we adopted the two-sample MR approach to assess the causal associations between four active herpesvirus infections (cold sores, mononucleosis, chickenpox, and shingles) and the risk of AD using summary statistics from GWAS, that is, to evaluate the effects of VZV, HSV-1, and EBV infection in AD risk.

## Methods

### Exposure GWAS data set

For infections, we used the GWAS summary statistics data from 23andMe cohort [12]. Individuals were included in this GWAS analysis using a set of strict self-reported questionnaires about their history of infection diseases to define phenotypes [12]. Participants were selected for having > 97% European ancestry as determined through an analysis of local ancestry. We focused on four infections (chickenpox, shingles, cold sores, mononucleosis) all caused by members of the human herpesvirus that were discussed in association with AD [1, 2]. The detailed descriptions (age, sex, sample size, etc.) of the GWAS data are presented in Table 1.

**Table 1:** Description of the GWAS studies used in the primary analysis

Variable/phenotype	Sample size (cases/controls)	Case mean/median AAO^a^	Control mean/median AAE^a^	% female, cases/controls	Phenotype ascertainment
AD GWAS (secondary analysis)	21,982/41944				
ADGC	14,428/14562	71.1	76.2	59.3/59.3	Autopsy or clinically confirmed from health care records
CHARGE	2137/13474	82.6	76.7	67.3/55.8	
EADI	2240/6631	75.4	78.9	65/60.6	
GERAD	3177/7277	73.0	51.0	64/51.8	
Herpesvirus infection GWAS					
Chicken pox	107,769/15982	45–60	45–60	51.9/37.0	Self-report questionnaires; vaccinated individuals were excluded from the controls in the chickenpox study
Shingles	16,711/118152	> 60	45–60	55.4/48.4	
Cold sores	25,108/63332	45–60	45–60	52.0/45.2	
Mononucleosis	17,457/68446	45–60	45–60	60.5/50.3	

^a^The 23andMe cohort did not provide the exact mean AAO or AAE in the infection GWAS; thus, we used the median age of their participants instead. *AAE* Age at examination or last follow-up, *AAO* Age at onset, *AD* Alzheimer disease, *ADGC* Alzheimer Disease Genetics Consortium, *CHARGE* Cohorts for Heart and Aging Research in Genomic Epidemiology Consortium, *EADI* The European Alzheimer’s Disease Initiative, *GERAD* Genetic and Environmental Risk in AD, *GWAS* Genome-wide association studies

### Outcome GWAS data set

In primary analysis, we used summary statistics data from a meta-analysis GWAS performed by International Genomics of Alzheimer's Project (IGAP) [11]. IGAP is a large two-stage study based upon GWAS on individuals of European ancestry. Data from stage 1 was used in the present study, including 63,926 individuals (21,982 AD cases and 41,944 cognitively normal controls) from four consortia. Summary details were given in Table 1. We additionally set out to validate our results in a family history of AD data set from UK Biobank [14]. Individuals with one or two parents with AD were defined as having family history of AD, which was ascertained via self-report. In this summary statistics data, an array of 314,278 participants in the UK Biobank were meta-analyzed, including 27,696 cases of maternal AD, 14,338 cases of paternal AD and 272,244 controls.

### Instrument identification

Only single nucleotide polymorphisms (SNPs) associated at genome-wide significance *P* value (*P* < 5 × 10^−8^) with a minor allele frequency greater than 0.01 were considered as potential instruments. Independent SNPs were selected at a threshold of linkage disequilibrium (LD) *r*^2^ > 0.05 and a distance of 1000 kb. For palindromic SNPs, we aligned strands using allele frequency and discarded palindromic SNP(s) that had minor allele frequency above 0.42. If any SNP was unavailable for an outcome summary data, we used LDlink (https://ldlink.nci.nih.gov/) API [15, 16] to identified proxy SNPs with a minimum LD *r*^2^ = 0.8. Then, exposure–outcome datasets were harmonized. We have considered the palindromic SNPs and checked original datasets to avoid reverse effects. We computed the proportion of variance in the phenotype explained by IV. The strength of the genetic instrument was judged by F-statistics**,** with a strong instrument defining as an F-statistic > 10 [17]. Lastly, we calculated the statistical power for this MR study with a two-sided type-I error rate *α* = 0.05 using R code provided by Burgess S [18]. The proportion of variance explained by IVs, F-statistics and power were presented in Additional file 1.

### Two-sample MR analysis

For each genetic instrument, the Wald ratio was calculated by dividing the effect size estimate for the association of the variant with the outcome by the corresponding estimate for the association of the variant with the exposure. When more than one SNPs were available, Wald estimates were meta-analyzed using inverse variance weighting (IVW) method. The IVW method will return an unbiased estimate in the absence of horizontal pleiotropy or when horizontal pleiotropy is balanced [19]. All causal estimates were converted to odds ratios (ORs) for the outcome was dichotomous. For exposure with more than three SNPs available (i.e., shingles), we conducted sensitivity analyses using weighted median [20], weighted mode [21], simple mode, MR Egger regression [22], and MR-PRESSO [23]. These methods hold different assumptions at the costs of reduced statistical power. The MR-Egger method is based on the “NO Measurement Error” (NOME) assumption (no measurement error in the SNP exposure effects), which is evaluated by the regression dilution *I*^2^_GX_ statistic (i.e., less than 0.9 indicates a violation of NOME) [24]. We used the MR Egger intercept, MR-PRESSO global test, and Cochran *Q* statistic to test the presence of heterogeneity or directional pleiotropy (i.e., shingles). Moreover, visual inspection of the forest plot, scatter plot, and leave-one-out plot were also used to assess the MR “no horizontal pleiotropy” assumption (see Additional file 6). For exposures with less than three SNP as IV (i.e., chickenpox, cold sores, mononucleosis), we performed another pleiotropy analysis using the Phenoscanner database to check significant associated traits of each SNP prioritized in the present study with phenotypes from previously published GWAS [25, 26].

To correct for multiple comparisons (four exposures), we applied a Benjamini–Hochberg false discovery rates (FDR) correction. *P* values below 0.05 but not survived the FDR correction were considered as suggestive of a potential association. Analyses were conducted using R version 3.6.3, with the MR analysis performed using the “TwoSampleMR” package version 0.5.2 [27, 28].

## Results

**Fig. 1:**
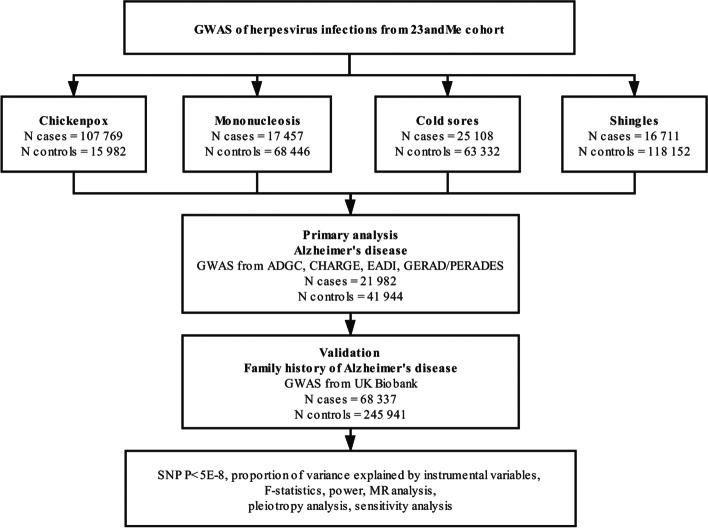
A flow diagram of the process in this Mendelian randomization analysis. AD, Alzheimer disease; ADGC, Alzheimer Disease Genetics Consortium; CHARGE, Cohorts for Heart and Aging Research in Genomic Epidemiology Consortium; EADI, The European Alzheimer’s Disease Initiative; GERAD/PERADES, Genetic and Environmental Risk in AD/Defining Genetic, Polygenic and Environmental Risk for Alzheimer’s Disease Consortium; GWAS, genome-wide association studies; N, number; SNP, single nucleotide polymorphism

A flow diagram depicting the process of the MR analyses is shown in Fig. 1. The 23andMe cohort identified one SNP for mononucleosis (rs2596465 in *HCP5* gene, *P* = 2.48 × 10^−9^), two SNP for chickenpox (rs10947050 in *RNF39*, *P* = 1.08 × 10^−10^; rs9266089 in *HLA-B*, *P* = 1.00 × 10^−10^), two SNP for cold sores (rs4360170 in *HCP5, P* = 3.41 × 10^−12^; rs885950 in *POU5F1*, *P* = 7.47 × 10^−13^), 15 SNPs for shingles in primary analysis and 13 SNPs in validation (Additional files 2, 3). Summary statistics for the genetic variants of herpesvirus infections and AD are presented in Additional file 3. Additional file 4 shows individual genetic estimates from each of the genetic variants in primary analysis and in validation.

**Fig. 2:**
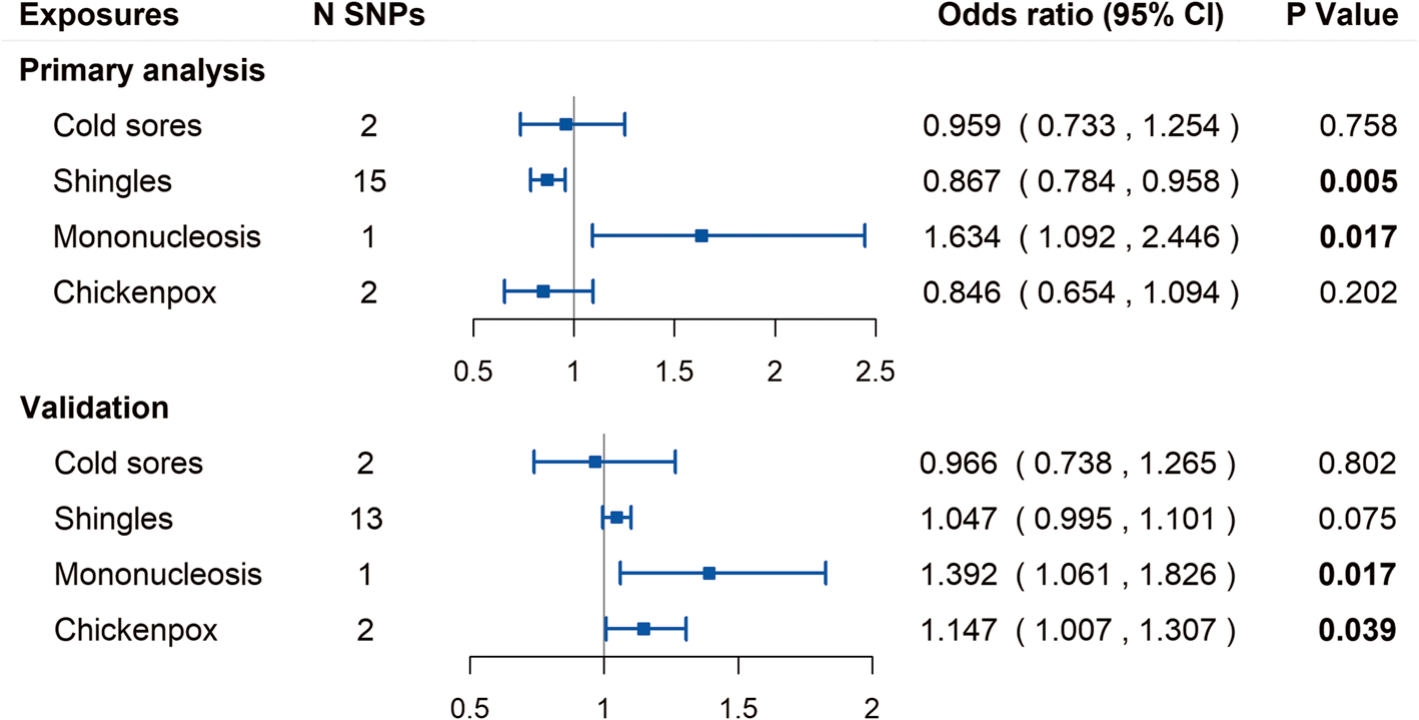
Forest plot of Mendelian randomization estimates for association between each herpesvirus infection and outcome. CI, confidence interval; N SNPs represents the number of single nucleotide polymorphisms used as instrumental variables

The estimates and 95% confidence interval (CI) for the Wald ratio or IVW analysis and the numbers of SNPs are presented in Fig. 2. In the primary analysis, Wald ratio showed that the genetically predicted mononucleosis was significantly associated with the risk of AD after FDR correction (OR [95% CI] = 1.634 [1.092, 2.446], *P* = 0.017, FDR-corrected *P* = 0.034). The result has been verified in UK Biobank GWAS and yielded similar patterns of effects with *P* value (OR [95% CI] = 1.392 [1.061, 1.826], *P* = 0.017).

Using IVW method, genetically predicted cold sores was not associated with risk of AD in primary analysis (OR [95% CI] = 0.959 [0.733, 1.254], *P* = 0.758) and was not associated with family history of AD in validation (OR [95% CI] = 0.966 [0.738, 1.265], *P* = 0.802). These findings concur with the results of the previous paper on HSV [8]. Genetically predicted chickenpox showed no association with AD (OR [95% CI] = 0.846 [0.654, 1.094], *P* = 0.202), but showed suggestive association with the family history of AD in validation (OR [95% CI] = 1.147 [1.007, 1.307], *P* = 0.039).

In primary analysis, IVW showed that the genetically predicted shingles were significantly associated with the decreased risk of AD (OR [95% CI] = 0.867 [0.784, 0.958], *P* = 0.005, FDR-corrected *P* = 0.020). Interestingly, the weighted median and MR-PRESSO methods support this significant association with *P* < 0.05. However, the estimate of association between shingles and family history of AD was marginally significant in validation (OR [95% CI] = 1.047 [0.995, 1.101], *P* = 0.075), and the point estimate direction was opposite to primary analysis. The Cochran *Q* statistic for shingles indicated no notable heterogeneity across instrument SNP effects (*P* > 0.05; see Additional file 2). The MR Egger intercept test (i.e., *I*^2^_GX_ = 0.98) and MR-PRESSO global test for shingles both suggested no horizontal pleiotropy (*P* > 0.05) in primary analysis and in validation. Additionally, for chickenpox, cold sores, and mononucleosis, we performed pleiotropy analyses by examining previously published GWAS to identified associated traits (see Additional file 5).

## Discussion

To our knowledge, this is the most comprehensive MR analysis to examine the causal associations of four herpesvirus infections and AD. Our results found a significant association between mononucleosis and AD, as well as an association between mononucleosis and family history of AD. The result is less susceptible to confounding and reverse causality bias than many previous conventional observational studies [29].

Mendelian randomization rests on three key assumptions [29]. The relevance assumption required that the genetic variants are robustly associated with the exposure of interest. We have selected our IVs from a large GWAS for infections. All SNPs were genome-wide significant (*P* < 5 × 10^−8^), which is a very strict threshold. Although the proportion of variance explained by IVs was not very high, the F-statistics were all highly above the threshold of weak instruments of F-statistic < 10 [17]. The other two assumptions are collectively known as independence from pleiotropy. In pleiotropy analyses, we found some SNPs (i.e., rs2596465, rs885950) were associated with “treatment with insulin” from UKB (see Additional file 4). However, recent MR analyses have suggested type-2 diabetes and high plasma glucose are not causally related to the risk of Alzheimer’s disease [30, 31]. According to the existing knowledge, there are no obvious evidences that SNPs in our study influence AD through other pathways, indicating our MR analysis to be valid.

The exposures in our MR analysis were defined by the self-reported history of infection diseases [12] rather than the serological or polymerase chain reaction (PCR) measures of exact pathogens which were often used in observational studies [32]. That was due to the lack of appropriate GWAS. However, researchers have done several surveys to define each infectious disease phenotype and have taken vaccination into account. And their surveys and phenotype scoring logic were showed in the original study [12]. From some point of view, defining infection by clinical diagnosis may have greater clinical significance for AD prevention and may provide a new perspective for exploring the mechanism of the causal effects.

We found evidence that mononucleosis (mainly caused by EBV) [13] was associated with a higher risk of AD. And it was validated using a GWAS dataset of the family history of AD, which enhanced the robustness of the causal relationship. As for EBV, a recent article detected EBV-specific T cell receptors in cerebrospinal fluid from patients with AD; however, their data were still not a directly evidence of a causality [6]. A meta-analysis based on two case–control studies demonstrates that the EBV infection (OR [95% CI] = 1.45 [1.00, 2.08]) is associated with a higher risk of AD [1]. A prospective cohort study also reports that the presence of EBV in the peripheral blood might be a risk factor for AD (OR = 1.843) [32]. Nevertheless, observational results are prone to reverse causation and confounding bias. Taking the advantage of overcoming these limitations adherent in observational studies [29], our MR findings can be used to provide more reliable evidence of causality between EBV and AD.

Although the specific mechanism underlying the association between infection and AD has not been fully understood, studies have proposed several possible mechanisms. Some have suggested that herpesviridae infection could promote the accumulation of amyloid-β plaques in brain [33]. Carbone et al. have suggested that persistent cycles of latency of the EBV might contribute to stress the systemic immune response and induce altered inflammatory processes, resulting in cognitive decline during aging [32]. Also, a recent article has found evidences indicating the effects of adaptive immunity in AD [6]. Our MR finding was from the aspect of mononucleosis other than the latent infection. In light of the fact that over 90% of the world’s adult population is chronically infected with EBV [34], our results from mononucleosis seem to be more practical, which might imply the underlying effects of immune mechanisms and provide contributions to the current literature [6].

There was no clear evidence to suggest an effect of chickenpox or shingles on AD. Although primary analysis showed a significant association between shingles and risk of AD, it was not validated in independent data, and the direction of point estimates in primary analysis and in validation analysis was the opposite. The two infection diseases are caused by VZV; however, chickenpox results from primary infection of VZV, while shingles are caused by the reactivation of the latent VZV within a dorsal root ganglion. Thus, the effect of the two infectious diseases on AD may be different. Although observational studies have found that VZV DNA and sera VZV antibodies showed no positive correlations with AD risk [35–37], it is not clear whether the primary infection and reactivation of VZV act differently on AD risk. In particular, recent cohort studies have reported that the use of antiviral agents in herpes zoster patients was associated with lower risks of dementia [38, 39], adding weight to the potential association between VZV infection and dementia. Further investigation is warranted concerning whether VSV reactivation is involved in triggering AD onset or progression.

Our MR results showed no significant association between cold sores, mainly caused by reactivation of herpes simplex virus type 1 (HSV-1), and AD risk. Accumulating evidence suggest HSV-1 alone does not confer an elevated risk of AD [35, 40, 41], but together with the carriage of *APOE*-ε4 allele increases AD risk [2, 42, 43]. Nevertheless, in an observational study which has determined *APOE* genotype and other possible confounders previously, they also suggested that both carriage of and reactivated HSV-1 infection increased the risk of developing AD [44]. A likely explanation for those controversial findings is that there could be some unmeasured confounding or other bias [3, 4]. A published MR study has suggested similar results as us that any HSV infection was not related to cognitive function or late-onset AD [8]. However, constrained by the MR approach, the potential link between APOE genotype and HSV-1 cannot be clarified in the present study, which requires further study. On the other hand, although the commonest manifestation of HSV-1 infection are cold-sores, only ~ 40% of people that are seropositive for HSV-1 actually get cold-sores. Therefore, cold sores are only a fairly specific subset of the infectious phenotypes that occur for HSV-1, and the effects of cold sores on AD are not equivalent to that of HSV-1 infection.

## Limitations

There are potential limitations to this study. First, some exposures have only one or two available SNP in our study, and the phenotypic variance tagged by SNP instruments was low (i.e., mononucleosis = 0.20%). However, it is unlikely to affect the statistical power for our MR analyses. Because the primary results are validated using an independent GWAS, and the sample size of datasets in validation is large enough to give a high power. Second, a general challenge of MR is the persistent possibility of horizontal pleiotropic associations between exposure and outcome. To avoid horizontal pleiotropy, we did pleiotropy analysis and checked phenotypes of each SNP. And based on current knowledge, we found no other associated traits were confirmed to have direct effects on AD. On the other hand, our results are less likely to be affected by pleiotropy and heterogeneity due to the small number of SNPs [7]. Third, participants of infection GWAS are limited to customer base of 23andMe, which may impact the MR results. And the self-reported information may lead to recall bias. Nevertheless, we did not find other appropriate infection GWAS to conduct MR analyses. Importantly, it should be noted that our analysis of infection refers to the infectious diseases caused by specific virus. Whether these present results are tenable in latent infection of those viruses is uncertain because of the different underlying pathologic changes. Moreover, as for mononucleosis, although 90% are caused by EBV, the remaining 10% is caused by other virus such as the human herpesvirus 6, which may limit our inference extended to EBV. Large and precise defined herpesvirus infection GWAS studies are needed to explore the MR application in this field.

## Conclusions

In conclusion, we found a positive association between mononucleosis and the risk of AD, as well as an association between mononucleosis and family history of AD from MR analysis. Further elucidation of this association could provide insights into the potential biological roles of mononucleosis in AD pathogenesis.

## Supplementary Information

**Additional file 1**. Mendelian randomization analyses of the association between herpesvirus infections and Alzheimer's disease.

**Additional file 2**. Sensitivity analysis, heterogeneity analysis and pleiotropy analysis of the association between shingles and Alzheimer's disease.

**Additional file 3**. Summary statistics for the genetic variants used to assess the effect of herpesvirus infections on Alzheimer's disease in the present Mendelian randomization study.

**Additional file 4**. Single SNP analysis of the association between mononucleosis, cold sores, chickenpox, shingles and Alzheimer's disease.

**Additional file 5.** Evidence of association (*p*<5×10-8) of significant SNP with other traits.

**Additional file 6**. Leave-one-out plots, forest plots, and scatter plots.

## Data Availability

All the data used in this study can be acquired from the original genome-wide association studies that are mentioned in the text. Any other data generated in the analysis process can be requested from the corresponding author.

## Abbreviations

AD: Alzheimer’s disease
EBV: Epstein-Barr virus
OR: Odds ratio
CI: Confidence interval
MR: Mendelian randomization
IV: Instrumental variables
GWAS: Genome-wide association studies
VZV: Varicella-zoster virus
HSV-1: Herpes simplex virus type 1
IGAP: International Genomics of Alzheimer's Project
UKB: UK Biobank
SNP: Single nucleotide polymorphisms
LD: Linkage disequilibrium
IVW: Inverse variance weighting
NOME: NO Measurement Error
MR-PRESSO: Mendelian randomization pleiotropy residual sum and outlier
