# Supplementary material for: Herpesvirus infections and Alzheimer’s disease: a Mendelian randomization study
Source: Alzheimers Res Ther. 2021 Sep 24;13:158. doi: 10.1186/s13195-021-00905-5 (PMC8464096; doi:10.1186/s13195-021-00905-5)
Supplement: Supplementary file 1 — Additional file 1. Mendelian randomization analyses of the association between herpesvirus infections and Alzheimer's disease. [file 13195_2021_905_MOESM1_ESM.docx]

**Additional file 1.** Mendelian randomization analyses of the association between herpesvirus infections and Alzheimer's disease

| Exposure (Herpesvirus infection) | N SNPs | Proportion of  variance  explained by IV | F-statistic | Power to  identify OR of 0.80 or 1.20 | Power to  identify OR of 0.70 or 1.45 |
| --- | --- | --- | --- | --- | --- |
| **Primary analysis** |  |  |  |  |  |
| Mononucleosis | 1 | 0.0020 | 175.16 | 0.17 | 0.52 |
| Cold sores | 2 | 0.0040 | 176.40 | 0.28 | 0.80 |
| Chicken pox | 2 | 0.0008 | 47.96 | 0.09 | 0.24 |
| Shingles | 15 | 0.0492 | 436.05 | 1.00 | 1.00 |
| **Validation** |  |  |  |  |  |
| Mononucleosis | 1 | 0.0020 | 175.16 | 0.48 | 0.97 |
| Cold sores | 2 | 0.0040 | 176.40 | 0.76 | 1.00 |
| Chicken pox | 2 | 0.0008 | 47.96 | 0.22 | 0.67 |
| Shingles | 13 | 0.0430 | 432.46 | 1.00 | 1.00 |

N SNPs represents the number of single nucleotide polymorphisms used as instrumental variables; IV, instrumental variable; F-statistic indicates the strength of the instrument variant used for each herpesvirus infection (a strong instrument is defined as an F-statistic >10).
