# Supplementary material for: Herpesvirus infections and Alzheimer’s disease: a Mendelian randomization study
Source: Alzheimers Res Ther. 2021 Sep 24;13:158. doi: 10.1186/s13195-021-00905-5 (PMC8464096; doi:10.1186/s13195-021-00905-5)
Supplement: Supplementary file 2 — Additional file 2. Sensitivity analysis, heterogeneity analysis and pleiotropy analysis of the association between shingles and Alzheimer's disease. [file 13195_2021_905_MOESM2_ESM.docx]

**Additional file 2.** Sensitivity analysis, heterogeneity analysis and pleiotropy analysis of the association between shingles and Alzheimer's disease

| **AD GWAS** | **N SNPs** | **MR method** | **OR (95%CI)** | **P_assoc_** | **P_pltr_** | **P_het_** |
| --- | --- | --- | --- | --- | --- | --- |
| Primary analysis | 15 | MR Egger | 0.847 (0.601, 1.192) | 0.358 | 0.891 | 0.108 |
|  |  | Weighted median | 0.874 (0.781, 0.978) | 0.019 | - | - |
|  |  | IVW | 0.867 (0.784, 0.958) | 0.005 | - | 0.146 |
|  |  | Simple mode | 0.907 (0.767, 1.072) | 0.270 | - | - |
|  |  | Weighted mode | 0.879 (0.755, 1.023) | 0.117 | - | - |
|  |  | MR-PRESSO | 0.875 (0.793, 0.965) | 0.018 | 0.154 | - |
| Validation | 13 | MR Egger | 1.054 (0.910, 1.220) | 0.500 | 0.929 | 0.087 |
|  |  | Weighted median | 1.068 (1.009, 1.131) | 0.024 | - | - |
|  |  | IVW | 1.047 (0.995, 1.101) | 0.075 | - | 0.122 |
|  |  | Simple mode | 1.064 (0.979, 1.158) | 0.171 | - | - |
|  |  | Weighted mode | 1.061 (0.988, 1.140) | 0.131 | - | - |
|  |  | MR-PRESSO | 1.096 (1.048, 1.146) | 0.001 | 0.989 | - |

AD, Alzheimer disease; GWAS, genome-wide association studies; CI, confidence interval; OR, odds ratio; P_assoc_, P-value for association; P_het_, P-value for heterogeneity between instrumental SNP causal estimates; P_pltr_, P-value for horizontal pleiotropy from MR-Egger intercept test or MR-PRESSO global test; N SNPs represents the number of single nucleotide polymorphisms used as instrumental variables; IVW, inverse variance weighted.
