# Supplementary material for: Herpesvirus infections and Alzheimer’s disease: a Mendelian randomization study
Source: Alzheimers Res Ther. 2021 Sep 24;13:158. doi: 10.1186/s13195-021-00905-5 (PMC8464096; doi:10.1186/s13195-021-00905-5)
Supplement: Supplementary file 3 — Additional file 3. Summary statistics for the genetic variants used to assess the effect of herpesvirus infections on Alzheimer's disease in the present Mendelian randomization study. [file 13195_2021_905_MOESM3_ESM.docx]

**Additional file 3.** Summary statistics for the genetic variants used to assess the effect of herpesvirus infections on Alzheimer's disease in the present Mendelian randomization study

| SNP | CHR | Gene | EA/NEA | MAF |  | Herpesvirus infections | | | |  | AD (primary analysis) | | | |  | Family history of AD (validation) | | | |
| --- | --- | --- | --- | --- | --- | --- | --- | --- | --- | --- | --- | --- | --- | --- | --- | --- | --- | --- | --- |
|  |  |  |  |  |  | Beta | SE | P value | N |  | Beta | SE | P value | N |  | Beta | SE | P value | N |
| Chickenpox |  |  |  |  |  |  |  |  |  |  |  |  |  |  |  |  |  |  |  |
| rs10947050 | 6 | RNF39 | T/C | 0.27 |  | -0.0904 | 0.0139 | 1.1E-10 | 123751 |  | 0.0172 | 0.0169 | 0.3097 | 63926 |  | -0.0155 | 0.0099 | 0.1181 | 314278 |
| rs9266089 | 6 | HLA-B | G/A | 0.85 |  | 0.1174 | 0.0180 | 1.0E-10 | 123751 |  | -0.0170 | 0.0216 | 0.4307 | 63926 |  | 0.0138 | 0.0098 | 0.1584 | 314278 |
| Cold sores |  |  |  |  |  |  |  |  |  |  |  |  |  |  |  |  |  |  |  |
| rs4360170 | 6 | HCP5 | G/A | 0.09 |  | -0.1449 | 0.0210 | 3.4E-12 | 88440 |  | -0.0067 | 0.0282 | 0.8132 | 63926 |  | -0.0065 | 0.0107 | 0.5436 | 314278 |
| rs885950 | 6 | POU5F1 | C/A | 0.42 |  | 0.0782 | 0.0109 | 7.5E-13 | 88440 |  | -0.0101 | 0.0151 | 0.5036 | 63926 |  | -0.0213 | 0.0100 | 0.0333 | 314278 |
| Mononucleosis |  |  |  |  |  |  |  |  |  |  |  |  |  |  |  |  |  |  |  |
| rs2596465 | 6 | HCP5 | T/C | 0.47 |  | 0.0729 | 0.0122 | 2.5E-09 | 85903 |  | 0.0358 | 0.0150 | 0.0174 | 63926 |  | 0.0241 | 0.0101 | 0.0166 | 314278 |
| Shingles |  |  |  |  |  |  |  |  |  |  |  |  |  |  |  |  |  |  |  |
| rs1130420 | 6 | HLA-DQB1 | G/A | 0.63 |  | -0.0780 | 0.0134 | 5.9E-09 | 134863 |  | 0.0547 | 0.0172 | 0.0015 | 63926 |  | - | - | - | - |
| rs114684640 | 6 | MICB | T/C | 0.04 |  | -0.1888 | 0.0312 | 6.2E-10 | 134863 |  | 0.0678 | 0.0343 | 0.0479 | 63926 |  | 0.0069 | 0.0096 | 0.4706 | 314278 |
| rs12528017 | 6 | - | C/A | 0.06 |  | 0.1700 | 0.0239 | 2.3E-12 | 134863 |  | -0.0178 | 0.0313 | 0.5706 | 63926 |  | 0.0114 | 0.0102 | 0.2667 | 314278 |
| rs12721829 | 6 | HLA-B | T/G | 0.26 |  | -0.1273 | 0.0202 | 2.4E-10 | 134863 |  | 0.0110 | 0.0202 | 0.5856 | 63926 |  | - | - | - | - |
| rs2023471 | 6 | TRIM31 | C/A | 0.59 |  | 0.0689 | 0.0123 | 2.1E-08 | 134863 |  | -0.0083 | 0.0153 | 0.5851 | 63926 |  | -0.0028 | 0.0100 | 0.7774 | 314278 |
| rs2523591 | 6 | HLA-B | G/A | 0.58 |  | 0.1344 | 0.0124 | 1.7E-27 | 134863 |  | -0.0220 | 0.0152 | 0.1491 | 63926 |  | 0.0017 | 0.0101 | 0.8675 | 314278 |
| rs2523815 | 6 | HCP5B | G/A | 0.36 |  | -0.1261 | 0.0130 | 1.8E-22 | 134863 |  | 0.0236 | 0.0157 | 0.1329 | 63926 |  | -0.0102 | 0.0100 | 0.3048 | 314278 |
| rs2763977 | 6 | - | G/A | 0.89 |  | 0.1157 | 0.0210 | 2.7E-08 | 134863 |  | -0.0069 | 0.0249 | 0.7829 | 63926 |  | 0.0219 | 0.0099 | 0.0264 | 314278 |
| rs28360997 | 6 | - | G/A | 0.83 |  | 0.1494 | 0.0169 | 3.1E-19 | 134863 |  | -0.0195 | 0.0200 | 0.3280 | 63926 |  | 0.0116 | 0.0101 | 0.2487 | 314278 |
| rs3095239 | 6 | TCF19 | G/A | 0.51 |  | 0.0727 | 0.0121 | 1.9E-09 | 134863 |  | -0.0061 | 0.0147 | 0.6781 | 63926 |  | -0.0256 | 0.0100 | 0.0107 | 314278 |
| rs41316748 | 6 | TNXB | T/C | 0.96 |  | -0.2161 | 0.0299 | 1.4E-12 | 134863 |  | 0.0101 | 0.0387 | 0.7941 | 63926 |  | -0.0141 | 0.0099 | 0.1535 | 314278 |
| rs7047299 | 6 | IFNA21 | G/A | 0.44 |  | -0.0693 | 0.0123 | 1.7E-08 | 134863 |  | 0.0005 | 0.0153 | 0.9716 | 63926 |  | -0.0065 | 0.0100 | 0.5121 | 314278 |
| rs77599976 | 6 | - | T/C | 0.13 |  | -0.1054 | 0.0187 | 1.3E-08 | 134863 |  | 0.0381 | 0.0228 | 0.0952 | 63926 |  | -0.0007 | 0.0098 | 0.9400 | 314278 |
| rs9260191 | 6 | HLA-A | G/A | 0.49 |  | -0.1007 | 0.0128 | 3.6E-15 | 134863 |  | 0.0273 | 0.0159 | 0.0850 | 63926 |  | - | - | - | - |
| rs9268557 | 6 | - | T/C | 0.51 |  | 0.0947 | 0.0121 | 4.3E-15 | 134863 |  | 0.0312 | 0.0151 | 0.0387 | 63926 |  | 0.0152 | 0.0100 | 0.1306 | 314278 |
| rs3130789 | 6 | LINC02570 | C/A | 0.59 |  | -0.0689 | 0.0122 | 1.8E-08 | 134863 |  | - | - | - | - |  | -0.0144 | 0.0101 | 0.1520 | 314278 |

AD: Alzheimer's disease; SNPs: single nucleotide polymorphisms; CHR: chromosome; SE: Standard error; N: sample size including cases and controls; EA: effect allele; NEA: non-effect allele; MAF, minor allele frequency.
