# Supplementary material for: Herpesvirus infections and Alzheimer’s disease: a Mendelian randomization study
Source: Alzheimers Res Ther. 2021 Sep 24;13:158. doi: 10.1186/s13195-021-00905-5 (PMC8464096; doi:10.1186/s13195-021-00905-5)
Supplement: Supplementary file 4 — Additional file 4. Single SNP analysis of the association between mononucleosis, cold sores, chickenpox, shingles and Alzheimer's disease. [file 13195_2021_905_MOESM4_ESM.docx]

**Additional file 4.** Single SNP analysis of the association between mononucleosis, cold sores, chickenpox, shingles and Alzheimer's disease.

| Study | SNPs | Traits | OR | LCI | UCI | P-value |
| --- | --- | --- | --- | --- | --- | --- |
| Primary analysis | rs4360170 | Cold sores | 1.047 | 0.715 | 1.534 | 0.812 |
|  | rs885950 | Cold sores | 0.879 | 0.602 | 1.283 | 0.504 |
|  | rs1130420 | Shingles | 0.496 | 0.322 | 0.764 | 0.001 |
|  | rs114684640 | Shingles | 0.698 | 0.489 | 0.997 | 0.048 |
|  | rs12528017 | Shingles | 0.901 | 0.628 | 1.292 | 0.570 |
|  | rs12721829 | Shingles | 0.917 | 0.672 | 1.252 | 0.586 |
|  | rs2023471 | Shingles | 0.887 | 0.574 | 1.370 | 0.587 |
|  | rs2523591 | Shingles | 0.849 | 0.680 | 1.060 | 0.148 |
|  | rs2523815 | Shingles | 0.829 | 0.650 | 1.059 | 0.133 |
|  | rs2763977 | Shingles | 0.942 | 0.618 | 1.436 | 0.782 |
|  | rs28360997 | Shingles | 0.878 | 0.675 | 1.141 | 0.330 |
|  | rs3095239 | Shingles | 0.920 | 0.619 | 1.367 | 0.678 |
|  | rs41316748 | Shingles | 0.954 | 0.672 | 1.356 | 0.794 |
|  | rs7047299 | Shingles | 0.993 | 0.644 | 1.530 | 0.974 |
|  | rs77599976 | Shingles | 0.697 | 0.456 | 1.064 | 0.095 |
|  | rs9260191 | Shingles | 0.763 | 0.560 | 1.039 | 0.086 |
|  | rs9268557 | Shingles | 1.390 | 1.017 | 1.900 | 0.039 |
|  | rs2596465 | Mononucleosis | 1.634 | 1.092 | 2.446 | 0.017 |
|  | rs10947050 | Chickenpox | 0.827 | 0.573 | 1.193 | 0.309 |
|  | rs9266089 | Chickenpox | 0.865 | 0.603 | 1.241 | 0.431 |
| Validation | rs4360170 | Cold sores | 1.046 | 0.905 | 1.209 | 0.544 |
|  | rs885950 | Cold sores | 0.762 | 0.593 | 0.978 | 0.033 |
|  | rs114684640 | Shingles | 0.964 | 0.873 | 1.065 | 0.472 |
|  | rs12528017 | Shingles | 1.069 | 0.951 | 1.203 | 0.264 |
|  | rs2023471 | Shingles | 0.960 | 0.722 | 1.276 | 0.779 |
|  | rs2523591 | Shingles | 1.013 | 0.874 | 1.173 | 0.866 |
|  | rs2523815 | Shingles | 1.084 | 0.928 | 1.267 | 0.308 |
|  | rs2763977 | Shingles | 1.208 | 1.022 | 1.429 | 0.027 |
|  | rs28360997 | Shingles | 1.081 | 0.947 | 1.234 | 0.251 |
|  | rs3095239 | Shingles | 0.703 | 0.537 | 0.921 | 0.010 |
|  | rs3130789 | Shingles | 1.232 | 0.925 | 1.643 | 0.154 |
|  | rs41316748 | Shingles | 1.067 | 0.976 | 1.168 | 0.154 |
|  | rs7047299 | Shingles | 1.098 | 0.828 | 1.457 | 0.516 |
|  | rs77599976 | Shingles | 1.007 | 0.839 | 1.208 | 0.943 |
|  | rs9268557 | Shingles | 1.174 | 0.955 | 1.444 | 0.129 |
|  | rs2596465 | Mononucleosis | 1.392 | 1.061 | 1.826 | 0.017 |
|  | rs10947050 | Chickenpox | 1.187 | 0.958 | 1.471 | 0.117 |
|  | rs9266089 | Chickenpox | 1.125 | 0.955 | 1.325 | 0.159 |

SNPs: single nucleotide polymorphisms; OR: odds ratio; LCI: lower 95% confidence interval; UCI: upper 95% confidence interval
