# Supplementary material for: Herpesvirus infections and Alzheimer’s disease: a Mendelian randomization study
Source: Alzheimers Res Ther. 2021 Sep 24;13:158. doi: 10.1186/s13195-021-00905-5 (PMC8464096; doi:10.1186/s13195-021-00905-5)
Supplement: Supplementary file 5 — Additional file 5. Evidence of association (p<5×10^-8^) of significant SNP with other traits. [file 13195_2021_905_MOESM5_ESM.docx]

**Additional file 5.** Evidence of association (p<5×10-8) of significant SNP with other traits.

| **Exposure** | **SNP** | **CHR** | **Gene** | **Trait** |
| --- | --- | --- | --- | --- |
| Mononucleosis | rs2596465 | 6 | HCP5 | Red cell distribution width (PMID: 27863252) |
|  |  |  |  | IgA deficiency (PMID: 27723758) |
|  |  |  |  | Allergic disease (PMID: 29083406) |
|  |  |  |  | Asthma (UK biobank) |
|  |  |  |  | Primary sclerosing cholangitis (PMID: 27992413) |
|  |  |  |  | Intestinal malabsorption or coeliac disease (UK biobank) |
|  |  |  |  | Self-reported psoriasis (UK biobank) |
|  |  |  |  | Treatment with insulin (UK biobank) |
|  |  |  |  | Rheumatoid arthritis (PMID: 24390342) |
| Cold sores | rs885950 | 6 | POU5F1 | Human Blood Cell (PMID: 27863252) |
|  |  |  |  | IgA deficiency (PMID: 27723758) |
|  |  |  |  | Diabetes diagnosed by doctor (UK biobank) |
|  |  |  |  | Intestinal malabsorption or coeliac disease (UK biobank) |
|  |  |  |  | Self-reported hyperthyroidism or thyrotoxicosis (UK biobank) |
|  |  |  |  | Self-reported psoriasis (UK biobank) |
|  |  |  |  | Treatment with insulin (UK biobank) |
|  |  |  |  | Unspecified hematuria (UK biobank) |
|  |  |  |  | Rheumatoid arthritis (PMID: 24390342) |
|  | rs4360170 | 6 | HCP5 | Human Blood Cell (PMID: 27863252) |
|  |  |  |  | Rheumatoid arthritis (PMID: 24390342) |
|  |  |  |  | Self-reported hypothyroidism or myxedema (UK biobank) |
|  |  |  |  | Treatment with levothyroxine sodium (UK biobank) |
| Chickenpox | rs9266089 | 6 | HLA-B | Human Blood Cell (PMID: 27863252) |
|  |  |  |  | Rheumatoid arthritis (PMID: 24390342) |
|  | rs10947050 | 6 | RNF39 | Human Blood Cell (PMID: 27863252) |
|  |  |  |  | Rheumatoid arthritis (PMID: 24390342) |
|  |  |  |  | IgA deficiency (PMID: 27723758) |
|  |  |  |  | Birth weight of first child (UK biobank) |
|  |  |  |  | Intestinal malabsorption (UK biobank) |
|  |  |  |  | Mouth or teeth dental problems: mouth ulcers (UK biobank) |
|  |  |  |  | Peak expiratory flow (UK biobank) |
|  |  |  |  | Self-reported malabsorption or coeliac disease (UK biobank) |
|  |  |  |  | Self-reported multiple sclerosis (UK biobank) |

CHR: chromosome; SNPs: single nucleotide polymorphisms.
