# Supplementary material for: Herpesvirus infections and Alzheimer’s disease: a Mendelian randomization study
Source: Alzheimers Res Ther. 2021 Sep 24;13:158. doi: 10.1186/s13195-021-00905-5 (PMC8464096; doi:10.1186/s13195-021-00905-5)

**Additional file 6 Leave-one-out plots, forest plots, and scatter plots**

**Supplementary Figure 1.** The leave-one-out plot, forest plot, and scatter plot for the association of shingles and Alzheimer’s disease in primary analysis.


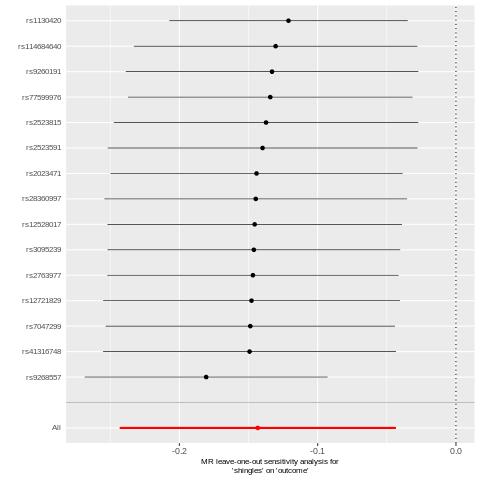


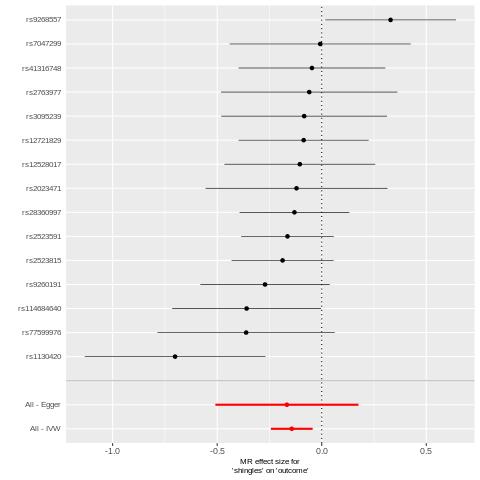


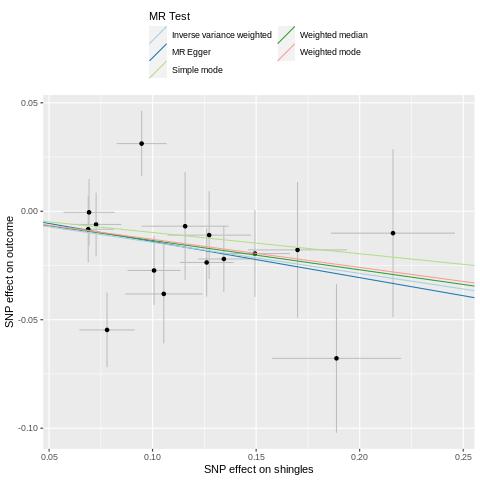


**Supplementary Figure 2.** The leave-one-out plot, forest plot, and scatter plot for the association of shingles and family history of Alzheimer’s disease in validation.


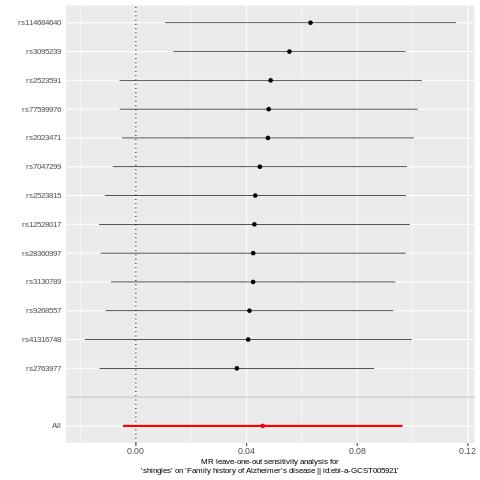

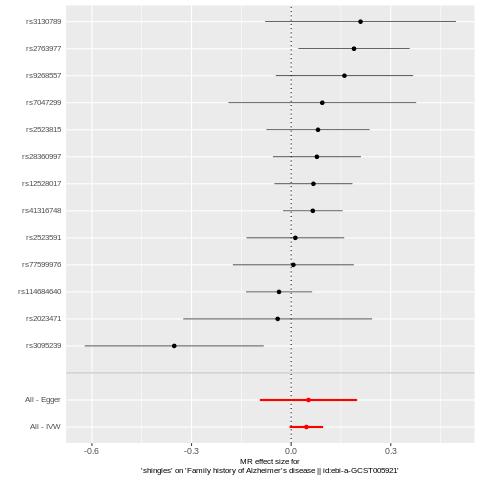


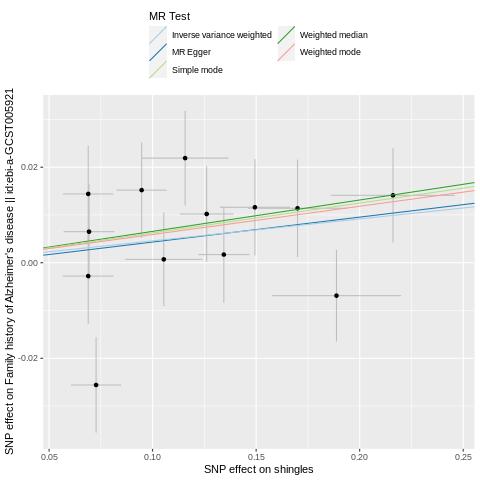


**Supplementary Figure 3.** The forest plot and scatter plot for the association of chickenpox and Alzheimer’s disease in primary analysis.


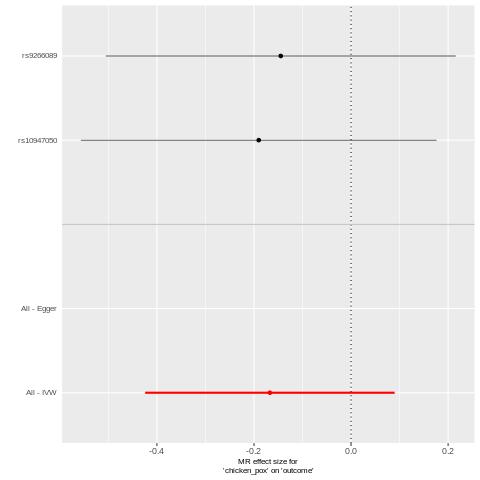

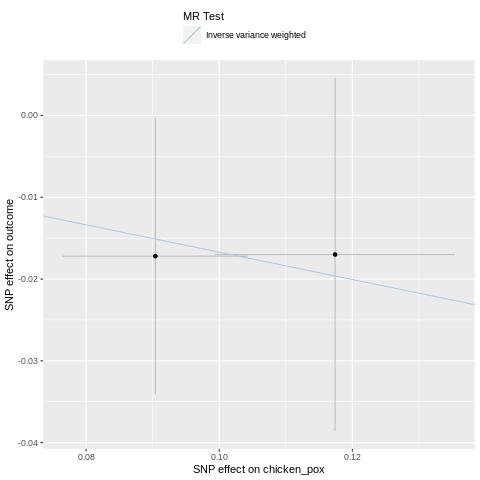


**Supplementary Figure 4.** The forest plot and scatter plot for the association of chickenpox and family history of Alzheimer’s disease in validation.


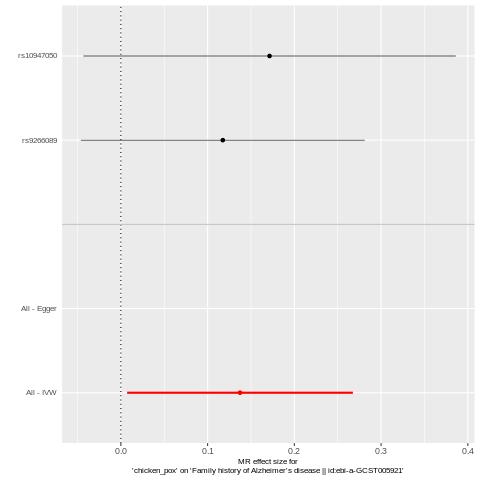


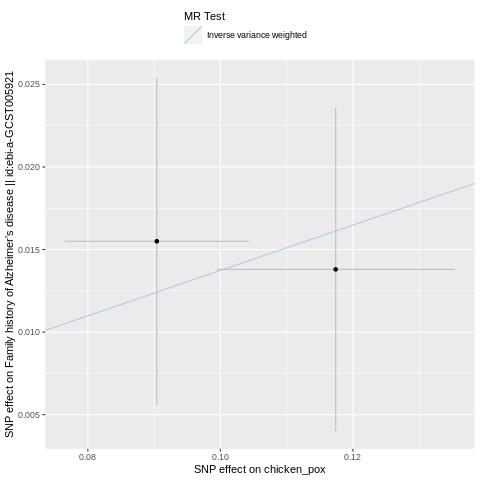


**Supplementary Figure 5.** The forest plot and scatter plot for the association of cold sores and Alzheimer’s disease in primary analysis.


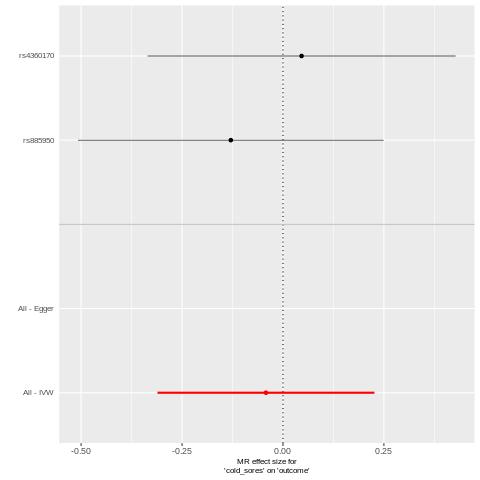


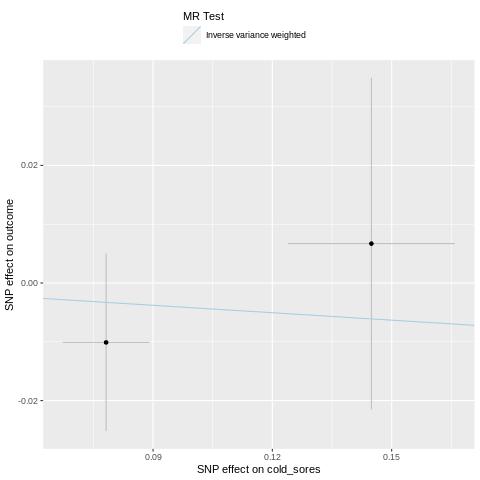


**Supplementary Figure 6.** The forest plot and scatter plot for the association of cold sores and family history of Alzheimer’s disease in validation.


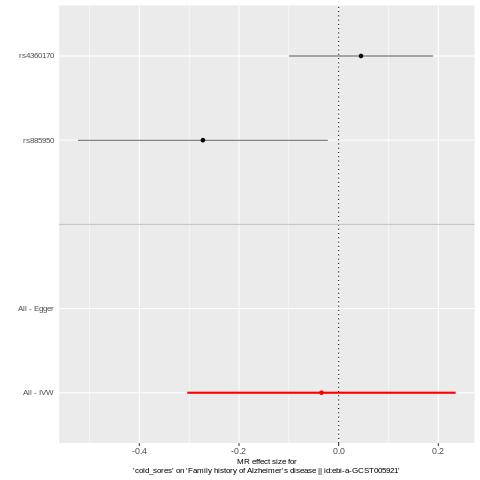


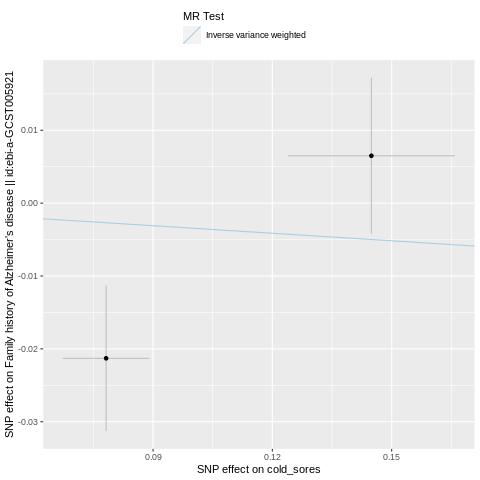

Supplement: Supplementary file 6 — Additional file 6. Leave-one-out plots, forest plots, and scatter plots. [file 13195_2021_905_MOESM6_ESM.docx]
